# Toward a Multimodal Computer-Aided Diagnostic Tool for Alzheimer’s Disease Conversion

**DOI:** 10.3389/fnins.2021.744190

**Published:** 2022-01-03

**Authors:** Danilo Pena, Jessika Suescun, Mya Schiess, Timothy M. Ellmore, Luca Giancardo, the Alzheimer’s Disease Neuroimaging Initiative

**Affiliations:** AbbVie, Alzheimer’s Association; Alzheimer’s Drug Discovery Foundation; Araclon Biotech; BioClinica, Inc.; Biogen; Bristol-Myers Squibb Company; CereSpir, Inc.; Cogstate; Eisai Inc.; Elan Pharmaceuticals, Inc.; Eli Lilly and Company; EuroImmun; F. Hoffmann-La Roche Ltd. and its affiliated company Genentech, Inc.; Fujirebio; GE Healthcare; IXICO Ltd.; Janssen Alzheimer Immunotherapy Research & Development, LLC.; Johnson & Johnson Pharmaceutical Research & Development, LLC.; Lumosity; Lundbeck; Merck & Co., Inc.; Meso Scale Diagnostics, LLC.; NeuroRx Research; Neurotrack Technologies; Novartis Pharmaceuticals Corporation; Pfizer Inc.; Piramal Imaging; Servier; Takeda Pharmaceutical Company; and Transition Therapeutics; ^1^Center for Precision Health, School of Biomedical Informatics, University of Texas Health Science Center, Houston, TX, United States; ^2^Department of Neurology, McGovern Medical School, University of Texas Health Science Center, Houston, TX, United States; ^3^Department of Psychology, The City College of New York, New York, NY, United States

**Keywords:** mild cognitive impairment, ADNI, longitudinal, deep learning, neuroimaging, clinical features, multimodal

## Abstract

Alzheimer’s disease (AD) is a progressive neurodegenerative disorder. It is one of the leading sources of morbidity and mortality in the aging population AD cardinal symptoms include memory and executive function impairment that profoundly alters a patient’s ability to perform activities of daily living. People with mild cognitive impairment (MCI) exhibit many of the early clinical symptoms of patients with AD and have a high chance of converting to AD in their lifetime. Diagnostic criteria rely on clinical assessment and brain magnetic resonance imaging (MRI). Many groups are working to help automate this process to improve the clinical workflow. Current computational approaches are focused on predicting whether or not a subject with MCI will convert to AD in the future. To our knowledge, limited attention has been given to the development of automated computer-assisted diagnosis (CAD) systems able to provide an AD conversion diagnosis in MCI patient cohorts followed longitudinally. This is important as these CAD systems could be used by primary care providers to monitor patients with MCI. The method outlined in this paper addresses this gap and presents a computationally efficient pre-processing and prediction pipeline, and is designed for recognizing patterns associated with AD conversion. We propose a new approach that leverages longitudinal data that can be easily acquired in a clinical setting (e.g., T1-weighted magnetic resonance images, cognitive tests, and demographic information) to identify the AD conversion point in MCI subjects with AUC = 84.7. In contrast, cognitive tests and demographics alone achieved AUC = 80.6, a statistically significant difference (*n* = 669, *p* < 0.05). We designed a convolutional neural network that is computationally efficient and requires only linear registration between imaging time points. The model architecture combines Attention and Inception architectures while utilizing both cross-sectional and longitudinal imaging and clinical information. Additionally, the top brain regions and clinical features that drove the model’s decision were investigated. These included the thalamus, caudate, planum temporale, and the Rey Auditory Verbal Learning Test. We believe our method could be easily translated into the healthcare setting as an objective AD diagnostic tool for patients with MCI.

## Introduction

Alzheimer’s disease (AD) is a progressive cognitive decline that severely disrupts activities of daily living. It is estimated that the number of people affected by AD will triple to over 120 million people by 2050, costing the United States alone billions of dollars in healthcare expenses (Lane et al., 2018). Further, no medications are currently available that can either reverse or stop the cognitive decline in subjects with AD. There is a clear need to develop novel treatments for those with AD. To accomplish this, early detection and identification of AD will facilitate the development of biomarkers and support the discovery of novel molecules by providing the right population for clinical trials.

Early dementia detection is paramount to decrease the chance of further comorbidities and mortality (Ahmed et al., 2019). This is especially relevant in clinical environments outside large academic centers, such as community hospitals, where resources are limited. Subjects with mild cognitive impairment (MCI) have many of the neurological deficits found in AD subjects. Additionally, about 10–15% of subjects with MCI will progress to AD every year (Plassman et al., 2008). This estimate is variable, with higher rates in clinical centers and some treatment trials; and lower numbers in population-based studies. Hence, subjects with MCI represent the perfect prodromal population for the exploration of conversion biomarkers, which has been one focus of the neurocognitive field (Desikan et al., 2010; Jack et al., 2010; Landau et al., 2010; Young et al., 2014; Liu et al., 2017; Ottoy et al., 2019; Giorgio et al., 2020). Creating a computer-assisted diagnosis (CAD) tool would provide an objective instrument for early AD diagnosis in patients with MCI. The vast majority of community hospitals can perform basic neuropsychological assessments and T1 magnetic resonance imaging (MRI); as such, we propose a multi-modal approach that combines both data sources to objectively and efficiently confirm the AD diagnosis in patients with MCI (which are at high risk of conversion).

For years, researchers have been investigating neuroimaging-based biomarkers in conjunction with computational tools to find early signs of AD within MCI subjects. Studies have looked at the differences between all of the combinations of healthy controls (CN), AD subjects, MCI subjects who have converted to AD (cMCI), and MCI subjects who have stayed stable (sMCI) (Mateos-Pérez et al., 2018). To determine the crucial features of an MCI subject which eventually converts to AD, we decided to focus on a cMCI vs. sMCI comparison. Current works have combined many types of data and a host of machine learning techniques. Recent papers have used T1-weighted MRI images and linear support vector machines (Sun et al., 2017; Tong et al., 2017), positron emission tomography (PET) and random forests (Nozadi et al., 2018), clinical information/neuropsychological measurements with ensemble learning (Grassi et al., 2019), and T1-weighted and diffusion MRI with linear models (Xu et al., 2019) to predict MCI conversion. However, many of these techniques require dimensionality reduction techniques, feature selection, lengthy image pre-processing pipelines, and other tabular data transformations that all require *a priori* hypotheses and increase the model and hyperparameter search space (Moradi et al., 2015; Ahmed et al., 2019).

Thus, scientists have turned to deep learning methods to abstract some of these steps that may incur bias throughout the pipeline. This class of models allows the incorporation of different types of data that form complex, non-linear relationships that could potentially provide more information about the conversion risk of an MCI subject. Some of the recent deep learning techniques for MCI classification use multimodal data types. These include T1-weighted MRI imaging with clinical variables (Spasov et al., 2019), cerebrospinal fluid imaging and longitudinal brain volumetric features (Lee et al., 2019a), T1-weighted and hippocampal imaging (Li et al., 2019), and a recurrent neural network (RNN) structure that uses cerebrospinal fluid, cognitive, and imaging biomarkers (Lee et al., 2019b). Using an array of data has been shown to have additive effects over using one data type alone for MCI classification. Researchers are also interested in developing a better understanding of the disease progression. Groups have predicted MCI clinical trajectories through a longitudinal feature framework (Bhagwat et al., 2018) and have used gray matter density maps at multiple time points as inputs to an RNN (Cui et al., 2019). This extension of data through time within one subject’s trajectory has proven a complicated but necessary problem to be able to incorporate all potential clinically available data (Lawrence et al., 2017). This is a non-exhaustive list of neuroimaging deep learning models for AD/MCI detection and prediction, and we refer to recent comprehensive reviews (Rathore et al., 2017; Ansart et al., 2021) for a complete list. This body of work focuses on the prediction of future AD in MCI subjects or diagnosis of AD using cohorts of subjects included in studies after their AD diagnosis, and therefore likely to have the disease for many years. To our knowledge, limited to no attention has been given to the development of automated CAD systems able to diagnose the conversion from MCI to AD, in patient cohorts followed longitudinally. This is important as these CAD systems could be used by neurologists and non-specialized physicians to monitor their MCI patients.

In our work, we propose to fill in this gap with a model that combines multi-modal longitudinal data that can be easily acquired in the vast majority of clinical settings in the industrialized world (e.g., T1-weighted magnetic resonance images, cognitive tests, and demographic information). This model is based on a compact convolutional neural network architecture that combines Attention and Inception modules which is computationally efficient and requires only linear registration between imaging time points. We test the conversion diagnosis performance of our model in a cohort of subjects that received a confirmed AD diagnosis after having MCI in a previous visit (cMCI) and subjects that remained with a stable MCI diagnosis (sMCI). Our dataset has a relatively large sample size (440 sMCI vs. 229 cMCI) compared to related methodological studies, which has been a common criticism (Mateos-Pérez et al., 2018).

## Materials and Methods

### Data

Data used in the preparation of this article were obtained from the Alzheimer’s Disease Neuroimaging Initiative (ADNI) database^1^ in October 2019. The ADNI was launched in 2003 as a public–private partnership led by Principal Investigator Michael W. Weiner, MD. The primary goal of ADNI has been to test whether serial magnetic resonance imaging (MRI), PET, other biological markers, and clinical and neuropsychological assessment can be combined to measure the progression of MCI and early AD.

Demographic information used in this study is displayed in Table 1. The majority of subjects were categorized as white (>93%) and non-Hispanic (>97%). Differences between sex counts were tested using Fisher’s exact test, and differences in baseline age, time between sessions, and years of education were evaluated with Wilcoxon rank-sum tests. *p*-Values of less than 0.05 were considered statistically significant.

**TABLE 1:** Demographics and time between imaging sessions of MCI subjects used in this study (1 SD).

	sMCI	cMCI	*p*-Value
Number of subjects	440	229	
Baseline age, years [mean (SD)]	73.4 (7.7)	74.2 (7.1)	0.167
Time between sessions, years [mean (SD)]	2.9 (2.2)	3.7 (1.9)	<0.0001
Years of education [mean (SD)]	15.8 (2.9)	15.8 (2.7)	0.831
Sex [male, *n* (%)]	260 (59.1)	134 (58.5)	0.934

For each subject, T1-weighted structural magnetic resonance images (MRI) were taken at two different time points in addition to clinical and demographic variables (age, sex, and years of education) available from ADNI. The clinical variables included APOe4 genotypes, neuropsychological cognitive tests like Montreal Cognitive Assessment (MoCA), Mini-Mental State Exam (MMSE), and the Dementia Rating Scale (CDRSB), the AD Assessment Scale (ADAS13, ADAS11, and ADASQ4), memory evaluations from the Rey Auditory Verbal Learning Test (RAVLT), and the functional activities questionnaire (FAQ). Additionally, we used AD and CN subjects to pre-train the model, and these subjects’ demographics are in Supplementary Material.

The time points used for cMCI subjects were chosen by selecting the session when the subjects were diagnosed with AD (session two) and the previous session where the subjects were still not converted (session one). Sessions for the subjects in the other cohorts (sMCI, AD, and CN) were chosen by selecting two consecutive sessions where both imaging and clinical evaluation were present. The current dataset did not allow a design to match the time between sessions for the whole cohort, this potential confounder is accounted for in our analysis.

Mild cognitive impairment conversion was clinically adjudicated by trained clinicians as described in the ADNI protocol. Any subject who converted back from AD to MCI was excluded from the study. Any subject included in the sMCI cohort remained stable for all sessions present in the ADNI dataset. For the subjects who converted (cMCI), the MRI images selected were based on the closest imaging session to the conversion adjudication; as such, we assumed that the T1 brain image would be representative of the status of the subject at the time of conversion as it is unlikely to significantly change in this time period. The average elapsed time between the time of conversion and the second imaging session was −0.7 ± 1.4 years.

The information on the conversion date can be found in the DXSYM_PDXCONV_ADNIALL.csv file from the ADNI database.

#### Image Preprocessing Pipeline

**FIGURE 1:**
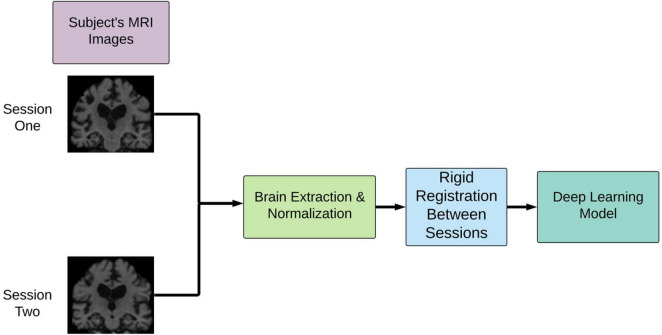
Overview of image pre-processing pipeline implementation. This pipeline involves rigid registration to align the patient’s brains intra-patient first and then inter-patient to a common space. cMCI subjects’ session one was before conversion, and session two was the imaging session at the clinically deemed conversion date. sMCI subjects, by definition, are not diagnosed with an AD conversion at any point.

As shown in Figure 1, the T1-weighted MRI images were pre-processed according to the steps outlined in our previous work (Pena et al., 2019). In summary, the two images at two time points were normalized and aligned to each other first and then registered to a common space using a linear registration algorithm. The normalization involved motion correction, non-uniform intensity normalization, and skull strip as implemented in the first pre-processing stages of the Freesurfer 6.0 pipeline. The final common interpatient space was derived from 2 mm MNI T1 template which was cropped of the background space to reduce the computational complexity of the network for a final resolution of 64 × 80 × 64. This pipeline was shown to drastically decrease the pre-processing time compared with conventional image processing pipelines such as the wull FreeSurfer-based ones (Pena et al., 2019). These steps were extended to the full MCI cohort used in this study.

Nine clinical variables were used at two different imaging sessions (e.g., cross-sectional variables). In addition, the longitudinal signed differences for each of these variables. Note that while the APOe4 genotype is not expected to change between sessions, it has followed the same processing for consistency and simplifying the evaluation of the feature importance. Age, sex, and years of education were also concatenated in the final feature vector used in the model. These clinical variables were all normalized by their mean value.

### Deep Learning Pipeline

#### Experimental Design

A 10-fold stratified cross-validation procedure was employed for model training and evaluation. Each fold was split into training, validation, and test sets with proportions of 80, 10, and 10%, respectively. Each fold maintained the distribution of sMCI/cMCI. Binary cross-entropy and the Adam were the loss function and optimizers used, respectively (Kingma and Ba, 2015). Each of the 10-folds had 75 epochs, and an early stopping condition of 10 epochs was implemented based on the model’s validation loss. Cyclical learning rates were used to dynamically change the learning rate throughout the training process (Smith, 2017). This method has been shown to potentially allow the model to “jump” out of local minima to subsequently find a lower minimum to reduce the overall loss. The upper and lower bounds for the learning rates were 1e−5 to 1e−8. A batch size of 4 was used in the experiments. The area under the receiver operating curves (AUC) and balanced accuracy were the experimental evaluation metrics. The DeLong’s test for statistical significance was used to test differences between AUC curves (DeLong et al., 1988). AUC curves’ 95% confidence intervals were calculated using a Monte Carlo resampling simulation with 1,000 iterations, and in each iteration, 80% of the total subjects’ probabilities were randomly chosen.

Two of the experiments used a transfer learning approach where an additional set of 190 AD and 243 CN subjects were first used to pre-train the network aimed at a simpler task first. None of the 433 AD/CN subjects in this pretraining step were used for the cross-validation, this avoided any risk of data leakage. The pretraining step followed all image pre-processing, hyperparameters, and initializations as stated in the text above, except for the cross-validation procedure. Finally, the pre-trained model was then used as the starting point for the weights used in the MCI classification task.

A fully connected network using only clinical variables was tested to obtain baseline comparison with the multi-modal network. This model is effectively equivalent to a logistic regression trained using the same optimization technique and validation approach as the multi-modal network; as such, it will allow for a fair evaluation of the relative improvement of adding brain imaging to the clinical data. The input feature vectors were the clinical variables, and the outputs were the same as the multi-modal network.

The experiments outlined were completed using Python 3.7, Keras version 2.2, and TensorFlow 1.14. The graphical processing units used were GeForce RTX 2080 Ti with 11 GB RAM. The training times varied between 30 and 90 s per epoch, depending on the architecture and experimental setup. The computational performance at inference time, which is more relevant to evaluate the ease of deployment of the model in a clinical environment, is discussed in section “Results.”

#### Deep Learning Architecture

The network architecture employed was inspired by a model that learned from spatial symmetry between brain hemispheres in the stroke detection task (Barman et al., 2019; Sheth et al., 2019). Our previous work extended this model in the AD-progression and time domain (Pena et al., 2019). This study has implemented a new network that combines cross-sectional and longitudinal imaging data with clinical features, which can be trained end-to-end on the MCI conversion classification task. Further, we focused our efforts on a less parameterized network to improve computational efficiency, as we are primarily concerned with the clinical application of this class of methods. This was possible through residual attention-based modules (Wang et al., 2017), allowing the network to focus on specific areas of the image with an Inception-based network, which leads to learning convolutional filters at different scales.

**FIGURE 2:**
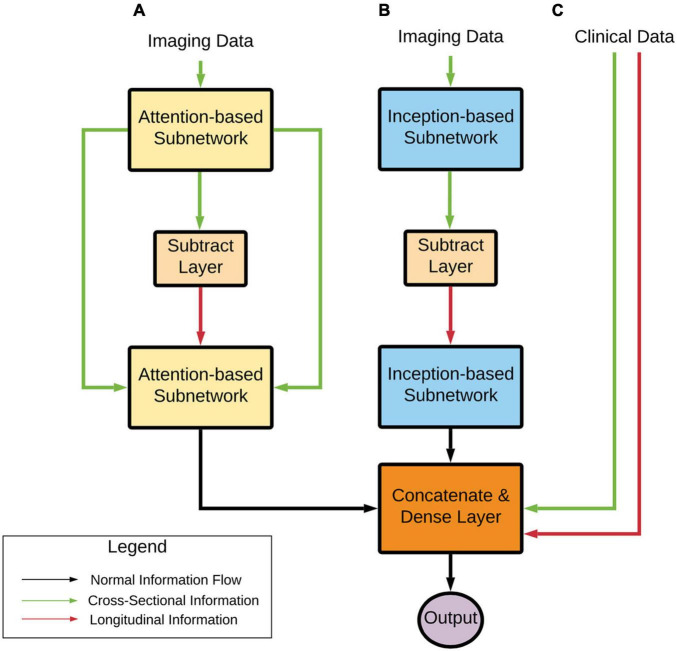
Deep learning architecture high-level overview. Imaging data pass through **(A)** an Attention-based subnetwork and **(B)** an Inception-based subnetwork. The Attention-based network includes skip connections that concatenate cross-sectional information to the processed longitudinal information. The Inception-based network only contains longitudinal information. These two subnetworks’ outputs are combined with clinical variables **(C)** that contain both cross-sectional and computed longitudinal differences. Finally, these subnetworks are combined and input into a prediction layer. Note that transfer learning approaches were used with AD and CN data, as stated earlier.

From a high level (Figure 2), the model can learn a complex representation of two images at different time points through the two subnetworks (Attention and Inception) in addition to the temporal differences of the two brains through the subtraction layer. This subtraction layer is sensitive to changes and is referred to as the “longitudinal” portion of the network. In the attention module, cross-sectional information is added through skip connections. The output from these two subnetworks is combined with clinical variables in a final dense layer for prediction.

This model has several benefits:

• It has the potential to identify structural changes in T1-weighted MRI scans over time, which is vital for determining MCI conversion while utilizing commonly available clinical information.
• It uses attention-based networks and deliberately leverages a less parameterized network that inherently regularizes the weights to only focus on important information.
• It incorporates an inception-based network that allows the model to use multi-resolution to represent the images at different scales in a non-sparse fashion.

#### Residual Attention Modules

Wang et al. (2017) extended the previously studied attention mechanism and applied it to their approach for image-level classification. Their overall network was composed of blocks named the residual attention module. These modules combined normal convolutional blocks (e.g., convolution, back normalization, and max pooling) with a U-Net inspired structure (Ronneberger et al., 2015) through a multiplication operator. The U-Net subunit allows the model to learn important information representing the input image through an encoder-decoder-like structure. The convolutional blocks allow the model to pay “attention” to these critical parts of the image through multiplication. This output then goes through another series of convolutional layers for further learning. Wang et al. (2017) stacked these residual attention modules to create a deep structure with complex attention mechanisms at different scales of the images. However, to create a less parameterized network, we limited the proposed network to just one residual attention block. For additional details about these modules, we refer the readers to the original publications.

#### Inception Modules

The inception modules used in this paper were inspired by the work done by Szegedy et al. (2015) and were extended to the 3-dimensional space (3D). The inception modules used were a combination of multi-resolution 3D convolutional layers. These layers were composed of three parallel operations: 1 × 1 × 1, 3 × 3 × 3, and 5 × 5 × 5 convolutions with two filters. Previous work has shown that this module can produce meaningful results in neuroimaging applications (Barman et al., 2019, 2020; Pena et al., 2019). This style of operation allows the model to view an image or input at different scales to learn different types of spatial information. The outputs were then concatenated and served as input to the next network layer. For additional details about these modules, we refer the readers to the original publications.

#### Layers

**FIGURE 3:**
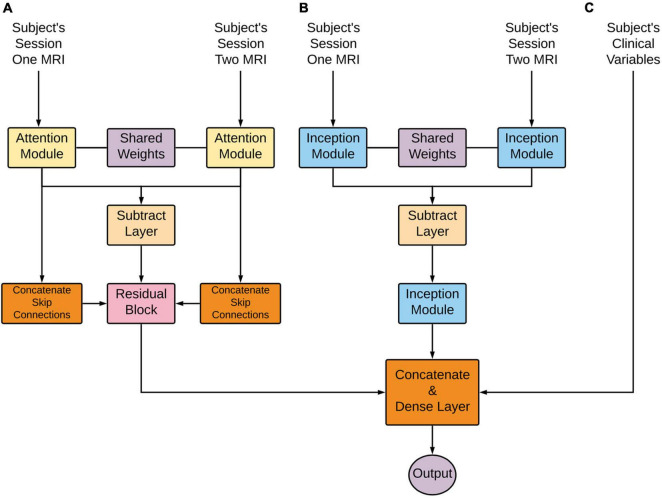
Deep learning architecture detailed overview. Longitudinal images pass through **(A)** an Attention-based network and **(B)** an Inception-based network. These subnetworks are composed of an initially shared weight representation, a subtraction layer, and a subsequent flatten layer. These two subnetworks’ outputs are combined with clinical variables **(C)**. Lastly, this concatenation is put through a dense layer for the final prediction.

From the overall network perspective (Figure 3 below), the first module layers learned a representation shared between the first and second imaging time points. This representation proceeded to a subtraction layer that took the difference between the two sessions, and this difference was the input to another module. These longitudinal outputs then went through another module to further learn from the differences between the two sessions. Note that the attention subnetwork incorporated longitudinal and cross-sectional information through the addition of skip connections, as seen in the figure below. Next, these outputs were flattened and concatenated with each other to form an imaging and clinical feature vector. Finally, the prediction layer was used for prediction utilizing the SoftMax activation function.

The code repository for this publication can be found at https://gitlab.com/lgianca/deepsymnet-att.

### Confounding Variable Adjustment

A logistic regression model was fit with the deep neural network’s probability output, baseline age, time between imaging sessions, and sex to adjust for any potential confounders inherent in the data chosen for the model. The logistic regression model coefficients, the 95% confidence intervals, and corresponding *p*-values were reported.

### Feature Importance

To develop an intuition about which voxels from the T1-weighted MRI images and features from the clinical variables, we employed the epsilon layer-wise relevance propagation (e-LRP) method (Bach et al., 2015). The e-LRP method starts from the prediction layer and works its way backward through the network. Layer-by-layer, the relevance of each of the previous layer’s nodes is computed until the operation reaches the input data layer. Each feature in the input data is assigned a final relevance score that describes how important that feature was for the final prediction. The codebase used in our experiments follows the implementation of DeepExplain (Ancona et al., 2018).

As stated above, relevance scores are projected onto the input data, which, in this study, are the two T1-weighted images and the subject’s clinical feature vector. We used a global and regional method to compute the magnitude of relative importance for the voxels in the MRI images. For the global method, each subject’s MRI relevance map was added for both sessions, and absolute values were used to remove the risk of canceling out relevance scores. Then, a heatmap allowed for the visualization of this global method.

For the regional method, the cortical and subcortical regions were segmented for each subject *via* the Harvard–Oxford atlas (Caviness et al., 1996). Then, for each subject and session, the summation of all the voxels’ magnitude in each region was calculated. This value was then divided by the volume of that particular region, resulting in a normalized relevance magnitude for a particular brain region. This final value allowed the different regions to be compared to one another on a similar scale. A similar approach was used to find the relative importance of the clinical features. The unsigned value for a clinical feature was added for each subject and then ranked in order of importance based on the magnitude of the total value. The overall method is described in greater detail in our previous work (Pena et al., 2019).

## Results

This study aims to (1) evaluate the use of different deep learning architectures, input data modalities, and transfer learning for MCI conversion classification using a computationally efficient architecture and to (2) investigate the important imaging and clinical features that drove the model’s decision based on the e-LRP method.

### Model Evaluation

**FIGURE 4:**
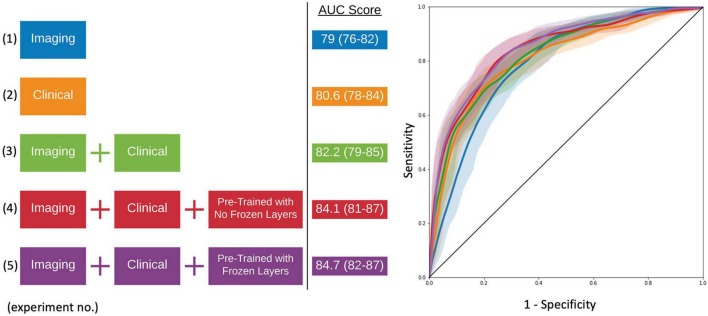
Model experiments and associated AUC scores by varying input data and the use of transfer learning **(*left*)**. ROC curves for comparing model performance from the experiments conducted **(*right*)**.

As seen in Figure 4 and Table 2, the model that used imaging and clinical input data was pre-trained using AD, and CN subjects with frozen weights had the highest AUC score (Experiment 5). This model was considered the “best” performing model in this paper. The pre-trained model where all of the weights could be fine-tuned had the best-balanced accuracy. Table 2 also shows that the improvement between solely using clinical variables (Experiment 1) and the best model that combined clinical and T1 imaging was statistically significant. Further, our best model was the only one significantly greater than the model that used clinical variables only. The average time taken to pre-process an image and for the model to make a prediction was 129.7 ± 19.8 and 0.12 ± 0.05 s, respectively.

**TABLE 2:**
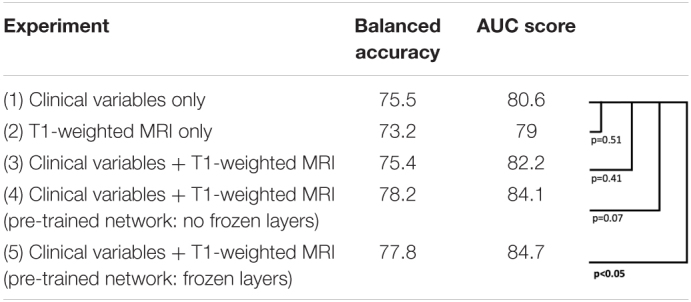
Model experiments’ metric comparison for balanced accuracy, AUC score, and testing for significant differences between AUC curves.

*p-Values were computed from the DeLong test for correlated ROC curves to reject the null hypothesis that there is no statistical difference between the AUCs.*

We evaluate the individual importance of the Inception and Attention subnetworks with ablation studies. We use as a base model and training strategy what has been described in Experiment 5. In order to account for the artificial advantage that the architectures might have solely on the basis of having more parameters, we increased the number of convolutional filters in each of the independent subnetworks to make them comparable with the full network. In Table 3, we show that the Inception-based subnetwork overperforms the Attention-based subnetworks. However, their combination (with the addition of the clinical data) outperformed the two architectures individually, even if the number of parameters was comparable.

**TABLE 3:** Ablation studies indicate that the combination of the two Attention and Inception-based subnetworks overperform the two individual subnetworks.

Experiment	Number of parameters	Balanced accuracy	AUC score
Attention-based subnetwork (imaging + pre-training with frozen layers)	374,398	65.7	68.6
Inception-based subnetwork (imaging + pre-training with frozen layers)	333,352	73.6	77.6
Best performing network (imaging + clinical + pre-training with frozen layers)	355,572	77.8	84.7

*Note that the number of convolutional filters in the subnetworks were increased to have comparable parameters with the entire network (i.e., within 10%).*

In order to evaluate the computational efficiency of the model, we evaluated the time required to generate a prediction at inference time (i.e., after model training) on an off-the-shelf laptop without using any GPUs. We repeated this 100 times and achieved an average execution time of 1.56 s (0.10 std). This does not take into account the file conversion, initial brain extraction and linear registration steps required, which can take from tens of seconds to a few minutes, depending on the software used. This compares favorably to the “*de facto*” Freesurfer-based longitudinal pipeline that can take an average of 17 h per subject (Pena et al., 2019) or methods relying on non-linear registration and extraction of the warp field, taking each image into template space. For example Spasov et al. (2019) report approximately 19,200 h of CPU time on a high-performance parallel computing cluster to non-linearly register the images, which is ∼19 h per subject.

### The Network as a Clinical Decision Support Tool

**FIGURE 5:**
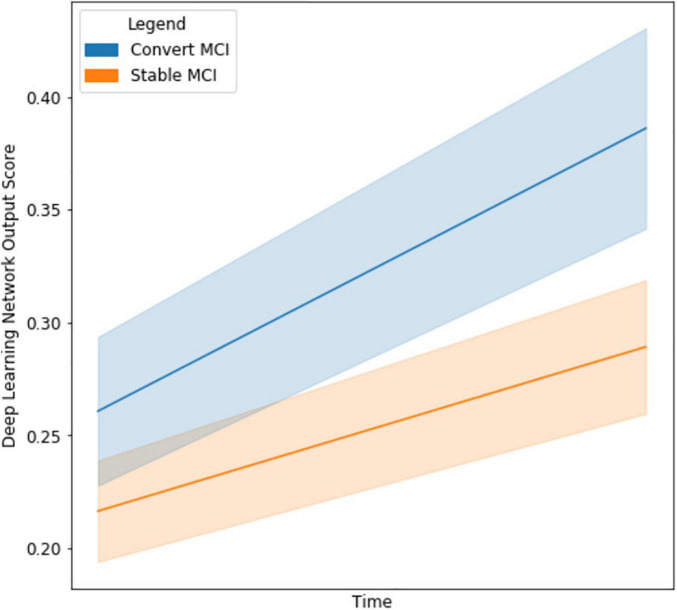
Line graph visualizing the difference in output network scores between cMCI (*blue*) and sMCI subjects (*orange*) with 95% confidence intervals in the shaded regions. The darker lines represent the mean trajectory based on the distribution of scores of the respective groups. Note that the cMCI subjects’ starting score is computed using the network and both imaging time points before conversion. The ending score includes the second time point when the AD conversion was diagnosed. The sMCI subjects’ starting scores are taken using the baseline and time point near the baseline date, and their ending score is using the baseline and a later date.

With the final model, we investigated the strength of the signal (deep network output probability) between MCI subjects who eventually converted to AD and those who stayed stable, as seen in Figure 5. The starting point for the cMCI subjects is higher than the sMCI subjects since there was some indication of AD conversion-like progression using MRI imaging time points before the actual conversion. However, this signal strengthens when an imaging time point around AD conversion is included, shown by the tendency toward higher probabilities on the right side of the figure. The sMCI group has a smaller slope with respect to time as there is no indication of AD conversion. This makes for a clear, qualitative difference between the two groups. The network derives a much stronger signal at the conversion point, indicating its ability to recognize patterns distinctly associated with AD conversion.

### Confounding Variable Adjustment

Further, the deep learning output probabilities were assessed for statistical significance with a logistic regression model and potential confounding variables. As seen in Table 4, the output probability remained statistically significant (*p* < 0.0001). Interestingly, though there were group differences between the imaging variable, as seen in Table 1, these differences were not significant when combined with the output probabilities.

**TABLE 4:** Summary of the logistic regression coefficients, confidence intervals, and *p*-values for model output probability and confounding variables.

Baseline age	Time between sessions	Years of education	Sex	Deep learning output probability
0.0140 (0.001–0.027)*	0.0916 (0.010–0.174)	0.0361 (−0.024 to 0.096)	0.0521 (−0.358 to 0.462)	−4.0714 (−4.700 to −3.443)***

*p < 0.05.

***p < 0.0001.

### Feature Importance

**FIGURE 6:**
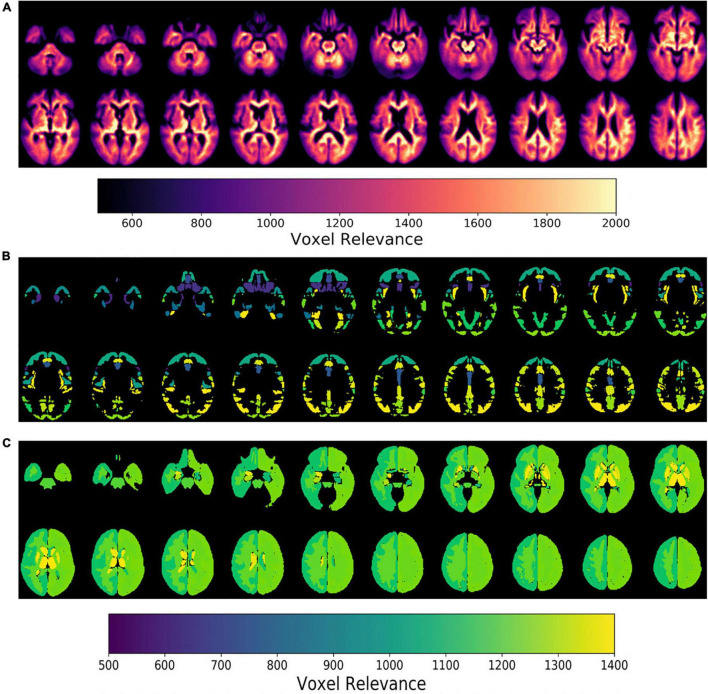
Epsilon layer-wise relevance propagation relevance maps displaying voxel-level and region-level contribution to model output probability at different brain slices. The maps are at the **(A)** voxel-level, **(B)** cortical, and **(C)** subcortical levels. Maps **(B,C)** are normalized to the brain region volume. The scales indicate the degree of voxel contribution magnitude.

Next, model feature importance was evaluated for both the imaging and clinical inputs. The imaging feature importance was completed on both a voxel-wise level and a brain regional level (subcortical vs. cortical regions), as seen in Figure 6. These saliency maps are smoother than our previous work (Pena et al., 2019), and we attributed this improvement to the use of attention-based modules and a less parameterized network. These model characteristics perform significant regularization, highlighting only the most informative regions for the given task.

**FIGURE 7:**
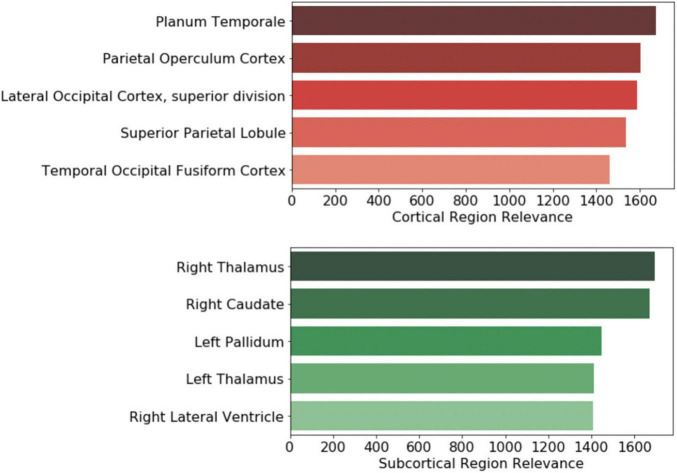
Brain regional contribution magnitude of the top five (*top*) cortical and (*bottom*) subcortical regions. The contributions were calculated by summating all the magnitudes within the brain region and then normalized to the brain region volume.

Further, the top five regions from the cortical and subcortical regions were plotted in Figures 6B,C. After volume normalization, the thalamus, caudate, pallidum, and lateral ventricle subcortical regions contained the highest overall contribution to the model’s decision. For the cortical regions, the planum temporale and parietal operculum cortex had the highest contributions. Both the cortical and subcortical regions had similar contribution magnitudes, as seen in the *x*-axis of Figure 7.

**FIGURE 8:**
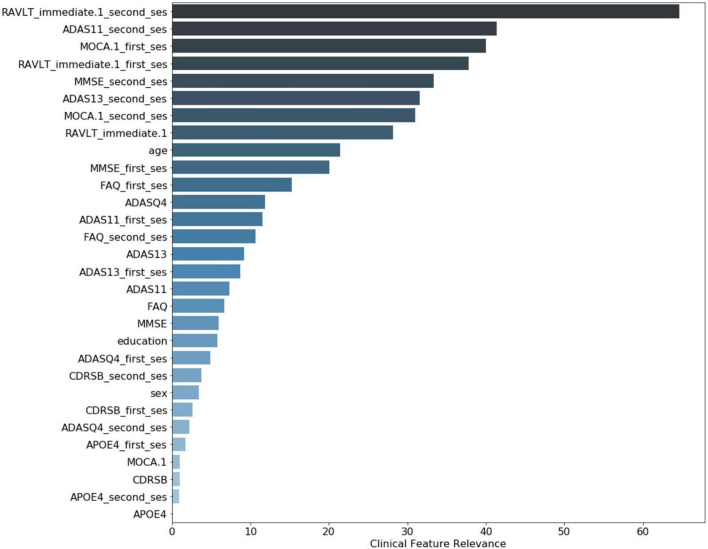
Clinical variable-level contribution magnitude. These values were calculated by summation of the contribution across all subjects. Neurological clinical variables denoted with “first_ses” and “second_ses” correspond to the subjects’ first and second clinical sessions, respectively. The clinical variables without a suffix represent the longitudinal change in that particular variable over time. Demographic variables included were age, sex, and years of education.

The clinical variables’ contributions are shown in descending order in Figure 8. The RAVLT score from the second session was the most important clinical feature, with ADAS11 from the second session and MoCA scores from the first session following.

## Discussion

This study employed a deep learning model to enable a CAD system able to provide an AD conversion diagnosis in an MCI cohort followed longitudinally. The model combined both Attention and Inception modules and was designed to be less parameterized to form a sparse yet rich representation of the input imaging and clinical features. The experiments performed demonstrated that the combination of imaging and clinical features produced a better model than using either type of data alone. Also, a model pre-trained on AD and CN subjects that served as a baseline for MCI classification was a better starting point for subsequent model fine-tuning than random weight initialization. Further, the brain regions that drove the model’s decision were visualized and quantified through the e-LRP method. The clinical features included in the model were also ranked and analyzed for relevance.

One of the main contributions of this network architecture is the combination of longitudinal and cross-sectional information. The subtraction operation was used between the two imaging and clinical time points and their respective features; thus, the network could learn from the differences over time. Further, this information was preserved throughout the training process by keeping the raw signal from the individual time points (e.g., cross-sectional data). The imaging-focused part of the network was divided into the Attention and Inception-based mechanisms. The attention module extended the residual attention used in computer vision, allowing the model to introduce sparsity into the network parameters. This allows the model to focus on certain parts of the brain input data related to MCI conversion to AD. The inception modules used 3D convolutional filters to find information at different spatial scales and granularity. We empirically show that using a combination of these modules, both the balanced accuracy and AUC were higher than using these modules individually in a network.

Further, we showed that the network improved AUC performance by incorporating more information in the time domain (cross-sectional and longitudinal) and in data modality (T1-weighted MRI and clinical features). This has been shown to be the case in related MCI and AD research (Goryawala et al., 2015; Spasov et al., 2019). The best model was also pre-trained on a cohort of AD and CN subjects. This model’s only trainable layer was the dense layer right before the prediction layer. Exclusively fine-tuning of the penultimate layer allowed the model to focus on changing a smaller number of weights compared to the entire model. This transfer learning setup also assumed that the brain representation from the AD and CN subjects was a good representation for an MCI application, making intuitive sense since this is modeling a progression pattern in subjects at high risk of developing AD in their lifetime. Finally, after controlling for several potential confounding factors, the network output probabilities remained statistically significant. Once the model is trained, the whole model can run in ∼1.5 s plus the time required to perform basic pre-processing involving file conversion, skull stripping, and linear registration (typically tens of seconds to a few minutes) on an off-the-shelf laptop without GPU. This would enable a neurologist to use this system as a computer-aided diagnostic tool during the office visit once the required imaging and/or clinical variables are acquired.

Once the experiments were completed, the crucial features for driving the model’s decision were investigated. To narrow down the imaging analysis for interpretation, we focused on the subcortical and cortical regions. The thalamus, caudate, pallidum, and lateral ventricle had the highest overall activation magnitude for the subcortical regions. Cholinergic synapses have a high density in several parts of the brain, including the thalamus, and have played a central role in research in aging and cognitive decline. Cholinesterase inhibitors are considered first-line treatments for mild and moderate AD (Hampel et al., 2018). Likewise, an *in vivo* imaging study found reduced serotonin transporter availability in MCI subjects in the thalamus compared to controls (Smith et al., 2017). Qing et al. (2017) found that impairment of spatial navigation skills, a clinical feature of AD found in MCI subjects, was significantly correlated to neuroimaging variable changes in the pallidum and thalamus.

Similarly, Fischer et al. (2017) investigated mobility changes in subjects with MCI. They found that decreased gray matter volume in the caudate nucleus was associated with a lower speed in functional mobility tasks. Crocco et al. (2018) applied a cognitive stress test to AD and MCI subjects and showed that negative clinical results were related to dilation of the lateral ventricle, among other regions. Yi et al. (2016) found that gray matter volumes in subcortical regions, including, but not limited to, the thalamus, caudate, and pallidum, were significantly reduced in MCI subjects when compared to controls. Additionally, many of these subcortical volumes were correlated with cognitive function.

For the cortical regions, the planum temporale, operculum cortex, and occipital cortex were some of the top regions with associated findings in AD and MCI literature. Researchers using several independent AD datasets found that anatomical changes in the planum temporale and thalamus were among the top features for their predictive model (Giraldo et al., 2018; Li et al., 2020). Others have found that changes in cortical minicolumn organization and premortem cognitive scores were significantly related in the planum temporale, potentially reflecting a phenomenon in brain atrophy in AD subjects (Chance et al., 2011). Alternatively, this might indicate the importance of auditory processing in MCI to AD progression as planum temporale is involved in auditory processing. A clinical study that used functional connectivity imaging and associated metrics found decreased intrinsic connectivity in the operculum cortex among MCI and AD subjects (Xie et al., 2012). Finally, a PET study demonstrated a significant and high overlap in hypoperfusion and hypometabolism in AD subjects in the occipital cortex (Riederer et al., 2018).

From the clinical features, ADAS, MoCA, and MMSE scores are among the top five variables with the most relevance for the model’s decision. This is unsurprising as MoCA and MMSE are the most widely used screening tools in clinical practice. ADAS is frequently used as a progression measurement in both clinical settings and clinical trials.

Interestingly, the RAVLT, a recent memory test, was the variable with the most relevance for the model’s decision for the first session, and it was ranked as one of the top five variables for the second session. Memory for recent events is distinctively impaired in AD and is served by the hippocampus, entorhinal cortex, and related structures in the medial temporal lobe.

This could indicate that the RAVLT provides more complementary information that is harder to directly learn from the imaging alone. Multiple clinical and neuroimaging studies have shown the importance of this variable in AD and MCI research; one of the earliest was performed by Estévez-González et al. (2003). More recently, Eliassen et al. (2017) used PET imaging and clinical scores to show that RAVLT were significant predictors in changes in cortical thickness between MCI and CN participants. A neuroimaging study conducted by Moradi et al. (2017) found that the MRI-based volumetric features were suitable variables for predicted parts of the RAVLT tool using an elastic net-based linear regression model. Russo et al. (2017) found that parts of the RAVLT assessment can have differences in discrimination accuracy and response bias between MCI and AD subjects, indicating there could be diagnostic specificity if using different test portions.

Our study has some limitations. First, the absolute classification performance of our method was lower than some found in the literature (Liu et al., 2017; Tong et al., 2017; Spasov et al., 2019) that report AUC scores around 90%. However, these models focus on the prediction of future AD rather than an actual diagnosis of the AD conversion, and they typically involve a very long pre-processing pipeline that would be hard to use in clinical settings. The use of longitudinal data to output an AD diagnosis can also be considered a limitation as it requires data from two-timepoints. The dataset used did include a majority of Caucasian non-Hispanic population, as such, the generalizability of the algorithm needs to be further confirmed on an entirely external dataset including a more diverse population. Finally, while the model can handle missing imaging or clinical data (as a whole), it currently cannot leverage clinical data with missing variables (unless imputation is used).

Future work could extend the ADNI dataset to incorporate multiple sources. This would increase the model’s generalizability to bias and errors that are inherent to different datasets. Also, adding more time points by extending this model using recursive neural networks or Gaussian processes algorithms could give a more nuanced trajectory signal that may unearth a strong signal for MCI progression and conversion to AD.

## Conclusion

In this paper, we introduce a novel method that utilizes T1-weighted MRI and clinical data at two-time points to diagnose AD in patients with MCI. At a high level, the model is a deep learning framework that combines residual Attention and Inception modules while taking advantage of cross-sectional and longitudinal data. The epsilon layer-wise propagation method allowed the interpretation of essential brain regions and clinical features that drove the model’s output. Some of the top subcortical and cortical regions included the thalamus, caudate, planum temporale, and operculum cortex. Further, RAVLT was the clinical feature that had the highest contribution to the final prediction. This method could easily be translated to the healthcare environment because it integrates variables commonly used in a clinical setting and has a fast image processing and prediction pipeline. This instrument could potentially be used as an objective and efficient diagnostic tool for patients at high risk of AD conversion.

## Data Availability Statement

Publicly available datasets were analyzed in this study. This data can be found here: adni.loni.usc.edu.

## Ethics Statement

Ethical review and approval was not required for the study on human participants in accordance with the local legislation and institutional requirements. The patients/participants provided their written informed consent to participate in this study.

## Author Contributions

DP, LG, MS, and JS contributed to conception and design of the study. DP performed the experiments and analysis, and wrote the first draft of the manuscript. DP, TE, and LG interpreted the results. All authors contributed to manuscript revision, read, and approved the submitted version.

## Conflict of Interest

The authors declare that the research was conducted in the absence of any commercial or financial relationships that could be construed as a potential conflict of interest.

## Publisher’s Note

All claims expressed in this article are solely those of the authors and do not necessarily represent those of their affiliated organizations, or those of the publisher, the editors and the reviewers. Any product that may be evaluated in this article, or claim that may be made by its manufacturer, is not guaranteed or endorsed by the publisher.

## Footnotes

1
adni.loni.usc.edu
