# Supplementary material for: Toward a Multimodal Computer-Aided Diagnostic Tool for Alzheimer’s Disease Conversion
Source: Front Neurosci. 2022 Jan 3;15:744190. doi: 10.3389/fnins.2021.744190 (PMC8761739; doi:10.3389/fnins.2021.744190)
Supplement: Supplementary file 1 [file Table_1.DOCX]

# Towards a Multimodal Computer-Aided Diagnostic Tool for Alzheimer's Disease Conversion

Danilo Pena (1), Jessika Suescun (2), Mya Schiess (2), Timothy Ellmore (3), Luca Giancardo (1), the Alzheimer's Disease Neuroimaging Initiative

(1) Center for Precision Health, School of Biomedical Informatics, University of Texas Health Science Center, Houston, TX

(2) McGovern Medical School, University of Texas Health Science Center, Houston, TX
(3) Department of Psychology, The City College of New York, NY

# Supplementary Materials

**Table 1a:** Subject demographics for AD and CN subjects used in transfer learning experiments.

|  | AD | CN | p-value |
| --- | --- | --- | --- |
| Number of subjects | 190 | 243 |  |
| Baseline age, years [mean (s.d.)] | 74.7 (7.8) | 74.5 (5.9) | 0.391 |
| Time between sessions, years [mean (s.d.)] | 1.6 (0.6) | 4.4 (2.5) | <0.0001 |
| Years of education [mean (s.d.)] | 15.1 (2.2) | 16.5 (2.7) | <0.0001 |
| Sex [male, n (%)] | 94 (49.5) | 125 (51.4) | 0.923 |
